# Age-related experiences of diverse older women living with HIV: A scoping review protocol informed by intersectionality

**DOI:** 10.1371/journal.pone.0306225

**Published:** 2024-06-26

**Authors:** Kristina Kokorelias, Paige Brown, Sharon Walmsley, Alice Zhabokritsky, Esther Su, Luxey Sirisegaram

**Affiliations:** 1 Division of Geriatric Medicine, Department of Medicine, Sinai Health System and University Health Network, Toronto, ON, Canada; 2 Department of Occupational Science and Occupational Therapy, Temerty Faculty of Medicine, University of Toronto, Toronto, ON, Canada; 3 National Institute on Ageing, Toronto Metropolitan University, Toronto, ON, Canada; 4 Rehabilitation Sciences Institute, Temerty Faculty of Medicine, University of Toronto, Toronto, ON, Canada; 5 Undergraduate Medical Education, Temerty Faculty of Medicine, University of Toronto, Toronto, ON, Canada; 6 Infectious Diseases, Department of Medicine, University Health Network, Toronto, ON, Canada; 7 Division of Infectious Diseases, Department of Medicine, University of Toronto, Toronto, ON, Canada; 8 CIHR Canadian HIV Trials Network, Vancouver, BC, Canada; Chinese Academy of Medical Sciences and Peking Union Medical College, CHINA

## Abstract

**Introduction:**

Older women living with HIV often go unnoticed due to societal biases and stigmas. Despite a rise in cases among older women, there is limited research on the psychosocial factors impacting their experiences. Aging complexities compounded by HIV and menopause affect these women’s health, while factors like mental health impact, changing support networks, and ageism with HIV stigma influence their well-being. Existing studies mostly compare older HIV-positive individuals without considering gender and intersectional identities, limiting understanding of their unique experiences. The scarcity of research addressing age-related differences from diverse perspectives delays the development of tailored treatments and interventions.

**Objectives:**

The study aims to comprehensively explore the age-related experiences of older women with HIV through three sub-questions that address (1) Key experiences, medical and social challenges, and strengths; (2) Impact of intersectional identities on their experiences; and (3) Gaps and limitations in current research.

**Methods:**

Utilizing a scoping review approach, the study seeks to map existing literature, employing a theoretical framework rooted in Sex- and Gender-Based Analysis Plus (SGBA+). Articles focusing on the age-related experiences of older women living with HIV aged 50 and above will be included. The study selection process will involve two independent reviewers screening articles based on pre-established inclusion criteria. Data extraction and synthesis will follow, analyzing the influence of sex, gender, and other identities on experiences.

**Discussion:**

The study’s comprehensive approach aims to bridge gaps in understanding older women’s HIV experiences, emphasizing intersectionality. While limited to English-language peer-reviewed articles, this review seeks to offer valuable insights for healthcare, policy, and research, potentially fostering positive change in the lives of diverse older women living with HIV.

## Introduction

Human Immunodeficiency Virus (HIV) is an infection which is often overlooked in populations of individuals who are 50 years of age or older [1]. This population may be overlooked due to societal bias surrounding aging, and HIV stigma for older adults [2–5]. According to the Public Health Agency of Canada, there have been 1,472 new HIV cases diagnosed in Canada alone in 2021, with women who are older adults comprising 17.6% of these cases [6]. Although there has been an increase in the number of older women living with HIV (for purposes of this paper, defined as women aged over the age of 50), there has been limited research focusing on the psychosocial factors that are associated with aging and living with HIV [7]. As such, the intersection of aging and HIV is a critical issue that necessitates a deeper understanding to better address the complex needs of older women living with HIV [1, 7–9].

The age-related experiences of older women living with HIV are often complicated by concurrent chronic health issues linked to the aging process and features of menopause [1, 10–14]. Additionally, the psychosocial well-being of older women living with HIV is influenced by factors such as the impact of HIV on mental health [15], changes in social support networks [16, 17] and relationships as women grow older [18, 19], and the intersection of ageism and HIV-related stigma [20, 21]. These factors collectively affect older women living with HIV’s subjective quality of life and overall well-being [22–24]. A scoping review on HIV, aging and health revealed that most of the existing literature involves the comparison of older individuals living with HIV to both younger individuals and those who were HIV-negative [25]. Additionally, the majority of the studies focus on individual experiences, regardless of gender [25]. While comparative studies are warranted, these comparisons may not be able to discern the unique role gender and other intersectional identities play in influencing age-related experiences of older women living with HIV. In response to this need, a scoping review explored the behavioral and psychosocial aspects of aging for older women living with HIV but noted little research examining age-related differences stemming from women’s race/ethnicity, socioeconomic status, sexual identity, and urban or rural location [7]. Consequently, this scarcity of research has led to delays in developing targeted treatment options and customized prevention and intervention programs for older women living with HIV [5].

Systemic factors such as sexism, racism, and poverty play a significant role in contributing to stigmatized identities among older women living with HIV [4, 5, 26]. These systemic factors intersect and reinforce one another, contributing to the formation of stigmatized identities and exacerbating the psychosocial challenges experienced by older women living with HIV. Sexism, entrenched within societal norms and structures, perpetuates the face double discrimination women living with HIV often face due to both their gender and HIV status [27]. This double discrimination often results in reduced health-seeking behaviours and missed medical appointments [28, 29] Additionally, racism exacerbates the stigma experienced by women of colour living with HIV, compounding the effects of discrimination and marginalization [29, 30], and increasing risk of depression [31]. Furthermore, poverty serves as a systemic barrier to healthcare, education, and social support networks for older women with HIV, thereby exacerbating the challenges they face in navigating their daily lives [32, 33].

An intersectional perspective recognizes that various factors can significantly impact the experiences of older adults, which is not always addressed in existing reviews. For example, the Durvasula (2014) [5] adopts a more traditional literature review approach, focusing solely on summarizing and synthesizing existing literature on HIV/AIDS in older women without consideration of their intersectional identities that influence the age-related experiences of older women living with HIV, including psychosocial challenges, intersectional identities, and existing research gaps identified in the literature. Intersectionality offers a vital framework to comprehend the interconnected identities and experiences of individuals [34]. Over the past three decades, intersectionality has gained prominence in diverse academic disciplines, particularly in feminist scholarship [35, 36]. Intersectionality underscores the significance of not approaching issues through a single-issue lens, emphasizing the intricate web of identities that profoundly affect individuals’ access to resources, their exclusion from social justice and political movements, and their day-to-day interactions [37–39].

In the context of older women living with HIV, it is crucial to recognize that the intersections of individual’s identities significantly influence their experiences, both in terms of privilege and discrimination [40, 41]. For instance, the needs and challenges faced by women of color living with HIV differ from those of other groups [42, 43]. Moreover, the intersectionality of aging with HIV as a female urges us to consider the multifaceted nature of stigmatized identities (i.e., being a women, living with HIV, ageism) [44]. A systematic review of existing reviews explored the intersectionality between HIV/AIDS, mental illness, and physical disability, but did not focus on older adults or women [44]. Therefore, to develop a comprehensive model for understanding how older women living with HIV, particularly those who may have multiple intersecting identities, navigate the health and social care system and their overall aging health, we must consider the complex interactions and challenges they face. This approach enables a more nuanced and holistic understanding of their experiences and informs more tailored and effective healthcare and support services.

### Objectives

In our endeavor to comprehensively explore the age-related experiences of older women living with HIV, we aim to answer the primary question of: What is the extent of the existing literature on the age-related experiences of older women living with HIV? To best address this we will answer three sub-questions:

1. What are the key experiences, medical and social challenges (e.g., comorbidity, stigma), and strengths of older women living with HIV (older women living with HIV)?;
2. How do intersectional identities (e.g., race, ethnicity, socio-economic status) impact the described experiences of older women with HIV?; and
3. What gaps and limitations exist in the current research related to older women living with HIV?

For the purpose of this study age-related experiences are defined broadly to include how the aging process interacts with HIV to influence health conditions, such as the increased risk of chronic illnesses or cognitive changes; the psychosocial well-being of older women, including the impact of HIV on mental health; shifts in social support networks and relationships as women grow older; the intersection of ageism and HIV-related stigma; barriers and facilitators related to healthcare access and quality of care as women age; the subjective quality of life and overall well-being in light of the aging process and living with HIV; sexual and reproductive health considerations specific to older age; and the availability and utilization of support services tailored to the unique needs of older women living with HIV.

This research encourages a shift toward more holistic, patient-centric, and evidence-based approaches, ultimately improving the well-being and healthcare outcomes of older women living with HIV. For healthcare providers, a deeper understanding of the multifaceted challenges faced by older women living with HIV can inform tailored and effective care. By recognizing the impact of intersectional identities and social determinants, providers can better address disparities and deliver patient-centered care. Policymakers can use the findings to inform and shape policies that promote equitable healthcare access, reduce stigma, and enhance support services for older women living with HIV. Additionally, researchers benefit from this study by gaining a foundation for future investigations that address identified gaps and limitations.

## Methods

### Design

A Scoping Review (ScR) has been deemed the most appropriate approach to address our research questions because it serves as a form of knowledge synthesis that is specifically designed to explore and map key concepts, various types of evidence, and research gaps within a defined area of study or clinical practice [45]. This is achieved through a systematic process of searching, selecting, and synthesizing existing knowledge [46]. ScRs have the advantage of encompassing diverse study types [46]. In our study, we will adhere to the ScR methodology framework originally outlined by Arksey and O’Malley [46] and subsequently refined by Levac et al. [47], Colquhoun et al. [48] and Peters et al. [49]. Additionally, we will follow the Preferred Reporting Items for Systematic Review and Meta-Analysis Protocols extension for ScRs statement, which provides guidelines for reporting ScRs effectively [50]. This protocol was also registered with Open Science Framework (Blinded for Review).

### Theoretical frameworks

As with other reviews involving older adults [51], we will adopt a theoretical framework rooted in the principles of Sex- and Gender-Based Analysis Plus (SGBA+) [52, 53]. SGBA+ was selected as it involves intersectionality frameworks and can therefore facilitate a nuanced examination of sample characteristics within the research processes and data, encompassing biological sex and the multiple social dimensions that older adults embody, such as ethnicity, income, age, race, education, and gender [51, 54, 55]. In the context of this review, women are defined based on the distinction between sex and gender. Sex refers to the biological construct and categorization of individuals as female, typically based on physical and physiological characteristics [51]. Gender encompasses the socially ascribed dimensions related to being female or male, taking into account the roles, expectations, and cultural attributes assigned to individuals within a specific society or culture [51]. Therefore, women in this context are individuals who are biologically categorized at birth as female (sex) and who identify with the social construct and roles associated with being women (gender).

#### Step 1: Research question

The previously outlined research questions were developed in consultation with our community advisory stakeholders through a workshop. We describe these stakeholders in a previous protocol (Blinded for Review). Our stakeholders include older women living with HIV and health and social care administrators.

#### Step 2: Identifying relevant literature

Following the guidelines recommended for ScRs and in consultation with an information specialist, we will develop an all-encompassing search strategy. In alignment with the review’s objectives, a set of key terms and Medical Subject Headings (MeSH) will be incorporated into the search, specifically targeting the categories of ’aged,’ HIV’ and ‘women’. The research strategy will be further refined through consultations with our stakeholders and research team (see S1 File for an example of a search strategy). The search will be limited to articles written or translated to English and human studies only. Stakeholders will be involved in the refinement of the search strategy, through virtual meetings to gather input and feedback. The final search strategy will be peer-reviewed following the PRESS guidelines [56]. We are proposing running the searches in the following databases: OVID Medline, Embase, PsychINFO, Emcare, and EBSCO CINAHL. We anticipate finalizing the search processes by June 2024. To ensure comprehensiveness, members of the research team will diligently review the reference lists of the studies that are included at the full text stage and actively seek input from stakeholders and experts to identify any additional relevant literature. All bibliographic information will be expertly managed using EndNote X8 [57], and systematic de-duplication will be executed according to the Bramer method for Endnote users [57]. Subsequently, records will be efficiently imported into Covidence, a web-based platform designed for screening and data extraction, streamlining the review process [58].

We will not include a search for grey literature in our review to maintain a streamlined focus on peer-reviewed, scientifically rigorous research sources, such that we can comment on the existing literature and make recommendations for future research.

#### Step 3: Study selection

We will include all peer-reviewed sources of quantitative, qualitative, mixed-method, or multimethod research, such that we are able to incorporate diverse types of evidence and research perspectives. To be eligible for inclusion, the participant sample of each study must consist of at least 50% older women aged 50 years and above who are living with HIV. Articles must focus on age-related experiences of the older women living with HIV participants. Articles must discuss either health related or social experiences. The participant sample must consist of at least 50% older women aged 50 years and above who are living with HIV. For mixed-gender studies to be included, they must present separate analyses or data specifically for older women in this demographic. In the context of HIV, health-related experiences will encompass the physical, mental, and emotional aspects of living with the virus or aging, including the management of symptoms, treatment adherence, and the impact on overall well-being. Social-related experiences involve the interactions, stigmatization, and support networks that individuals with HIV encounter, highlighting the influence of societal attitudes, disclosure, and access to social services on their lives. Due to resource constraints, only articles published in English will be included.

To ensure the consistency and reliability of the study selection process, two independent reviewers will apply the abovementioned pre-established inclusion criteria to all articles. Initially, the two reviewers will screen titles and abstracts to identify potentially relevant articles, followed by a thorough full-text review of the selected articles using the Covidence platform. Prior to the commencement of the screening process, all team members will meet to discuss the inclusion criteria and ensure the team is aligned in their understanding of the inclusion criteria. Additionally, weekly meetings will be conducted to address any ongoing concerns or uncertainties. In cases where there is a discrepancy or disagreement between the two reviewers during the selection process, consultation with a third team member will be initiated to facilitate a consensus decision.

#### Step 4: Data extraction

All studies and reports meeting the inclusion criteria will be systematically charted by two independent reviewers using the Covidence platform. To ensure consistency and reliability, a pilot of the data extraction form will be conducted on five randomly- selected studies. The articles will be selected using the random number generator in Microsoft Excel. The findings from this pilot process will be used to refine the data extraction form and ensure all relevant information is being captured consistently. Any discrepancies or challenges encountered during the pilot will be discussed among the research team, and the form will be revised as needed before proceeding with full data extraction on all included studies. As advocated by Arksey and O’Malley [46], the charting process is iterative, allowing for necessary form adjustments, which will be deliberated upon in consultation with the research team prior to extracting data from all included articles. The extracted data will encompass various elements, including publication type (e.g., journal article), study type (e.g., quantitative, qualitative, or mixed), study characteristics and settings, characteristics of the older women living with HIV (e.g., demographic [i.e., sex, gender, and other identity constructs [55]] and clinical attributes), including the intersections investigated (e.g., the specific intersections investigated in each study; e.g. race/ethnicity and gender), criteria and strategies employed for participant recruitment, results of the study, including how intersectionality was operationalized (e.g. use of interaction terms, stratification), the direction of any statistically significant intersectional inequality and how it was determined. To enhance the quality and accuracy of the data, a third reviewer will review and verify all extracted information. This comprehensive data collection process is anticipated to extend over a period of approximately 3 months.

#### Step 5: Data synthesis and presentation of results

The results of this review will be systematically presented in alignment with each research question. Each research question will be answered using a quantitative summary providing an overview of the quantity and types of included literature and a narrative synthesis that maps and interprets the findings. This narrative mapping will serve as the foundation for constructing the gaps and limitations in research (study question 3). Findings will be interpreted through an intersectional lens, highlighting how the intersection of multiple identities like race, gender, class shapes experiences in compounding ways that go beyond looking at these factors in isolation. The narrative synthesis will not only map the findings but also interpret them through an intersectional lens, highlighting how multiple identities intersect to shape experiences and outcome [59]. Where data is available, the analysis will aim to identify the influence of sex (biological attributes), gender (socially constructed roles) and other intersectional identities on experiences, and findings will be combined according to the intersections identified, for example, the intersection of race/ethnicity and genders. Results will be synthesized by mapping how different intersectional positions (e.g. Black women, low-income transgender women) are associated with disparities in experiences and outcomes related to living with HIV. All investigator members of the team, as well as representatives of the stakeholder advisory committee will be involved in discussing the final results and implications. Ongoing communication and scheduled meetings will facilitate this engagement. Regular consultations with stakeholders will be held monthly to review progress, discuss emerging findings, and provide feedback on data extraction and analysis. These consultations will take place through video conferences and email communications.

#### Step 6: Consultation and knowledge translation

We will encompass integrated knowledge transition (KT) and end-of-project KT strategies [60]. The research team will actively disseminate preliminary findings through presentations to local clinical and stakeholder audiences throughout the project, such that we can obtain preliminary feedback on themes. We will incorporate these findings into the preparation of a manuscript and subsequent conference presentation. The overarching goal of our KT plans are to create a strong evidential foundation that underpins the advancement of person-centered and integrated healthcare and social service delivery for older women living with HIV. This objective is driven by the overarching aim of improving the overall well-being and quality of life for older women living with HIV.

## Discussion

This scoping review will employ a comprehensive and systematic approach to map the existing literature on the age-related experiences of older women living with HIV. By considering a wide range of study types we will ensure diversity in the types of evidence examined. Moreover, the review is theoretically informed by an SGBA+ lens, which emphasizes the significance of considering intersectionality in research. This approach allows for an in-depth examination of how sex, gender, and other identity factors influence the experiences of older women with HIV. The engagement of diverse stakeholder groups, including researchers, older women living with HIV, clinical professionals, and health and social service decision-makers, ensures that the research questions, methodology, and KT strategies are inclusive and reflect the real-world perspectives of those affected by HIV. However, the review is limited by its inclusion of only English-language peer-reviewed articles. Despite efforts to include a diverse range of literature, some experiences of older women living with HIV, particularly those from underrepresented backgrounds, may not be adequately addressed in the existing English-language peer-reviewed literature. This constraint may introduce a language bias, potentially excluding relevant studies published in other languages. Recognizing this limitation is crucial, as it may affect the comprehensiveness and generalizability of our findings. To mitigate this bias in future research, we recommend considering the inclusion of non-English studies with translation support. This approach could enhance the diversity and comprehensiveness of the evidence base, ensuring that valuable insights from non-English publications are not overlooked. Nonetheless, this review will serve as a critical step in recognizing the unique challenges and strengths of older women living with HIV, offering valuable insights for healthcare providers, policymakers, and researchers. Through ongoing collaborative efforts and KT, we aim to bridge the gap between research and practice, fostering positive change in the lives of diverse women affected by HIV at older age.

As this is a scoping review synthesizing data from previously published literature, no direct involvement of human participants took place. Therefore, participant informed consent was not applicable. All data extracted and analyzed during this scoping review will be made publicly available as supplemental files upon publication. The dataset will include the full list of included studies and their characteristics, as well as the extracted data on intersectional identities, inequalities, and other relevant findings.

## Conclusion

In conclusion, the forthcoming scoping review will address the critical gap in understanding the age-related experiences of older women living with HIV. Despite the increasing prevalence of HIV among this demographic, research on their psychosocial challenges and healthcare needs remains limited. By systematically mapping and synthesizing existing literature, we aim to illuminate the complex intersection of aging, gender, and HIV, offering insights into the unique challenges faced by older women living with HIV. Our theoretical framework rooted in Sex- and Gender-Based Analysis Plus (SGBA+) will ensure a nuanced examination of how social identities intersect to shape experiences. Through collaboration with diverse stakeholders and rigorous knowledge translation strategies, this scoping review protocol will inform a review that aims to inform evidence-based interventions, policies, and healthcare practices tailored to the needs of older women living with HIV. Ultimately, our efforts seek to improve the overall well-being and quality of life for this marginalized population, fostering positive change in healthcare delivery and societal attitudes towards aging and HIV.

## Supporting information

S1 Checklist PRISMA-P (Preferred Reporting Items for Systematic review and Meta-Analysis Protocols) 2015 checklist: Recommended items to address in a systematic review protocol*. (DOC)

S1 File Sample search strategy. (DOCX)
