# Supplementary material for: Age-related experiences of diverse older women living with HIV: A scoping review protocol informed by intersectionality
Source: PLoS One. 2024 Jun 26;19(6):e0306225. doi: 10.1371/journal.pone.0306225 (PMC11207145; doi:10.1371/journal.pone.0306225)
Supplement: S1 File — (DOCX) [file pone.0306225.s002.docx]

OVID Medline

Ovid MEDLINE(R) ALL <1946 to December 07, 2023>

1 hiv/ or hiv-1/ or hiv-2/ 108287

2 hiv infections/ or acquired immunodeficiency syndrome/ or acute retroviral syndrome/ or aids arteritis, central nervous system/ or aids dementia complex/ or aids-associated nephropathy/ or aids-related complex/ or aids-related opportunistic infections/ or hiv enteropathy/ or hiv seropositivity/ or hiv wasting syndrome/ or hiv-associated lipodystrophy syndrome/ 319079

3 (HIV or (human adj1 immunodeficien*) or (human adj1 immune adj1 deficien*)).tw,kf,kw. 381075

4 ((AIDS adj1 virus) or (acquired immune adj1 deficien*) or (acquired adj1 immunodeficien*)).tw,kf,kw. 29060

5 1 or 2 or 3 or 4 457907

6 aged/ or "aged, 80 and over"/ or centenarians/ or nonagenarians/ or octogenarians/ or frail elderly/ or middle aged/ 5586516

7 ((Old adj2 Adult*) or (Old adj2 Person*) or (Old adj2 People*) or (Old adj2 Patient*) or (Old adj2 Citizen*) or (Older adj2 Adult*) or (Older adj2 Person*) or (Older adj2 People*) or (Older adj2 Patient*) or (Older adj2 Citizen*) or (Oldest adj2 Adult*) or (Oldest adj2 Person*) or (Oldest adj2 People*) or (Oldest adj2 Patient*) or (Oldest adj2 Citizen*) or (Ag?ing adj2 Adult*) or (Ag?ing adj2 Person*) or (Ag?ing adj2 People*) or (Ag?ing adj2 Patient*) or (Ag?ing adj2 Citizen*) or (Aged adj2 Adult*) or (Aged adj2 Person*) or (Aged adj2 People*) or (Aged adj2 Patient*) or (Aged adj2 Citizen*) or (Middle adj1 Age) or (Middle adj1 Aged)).tw,kf,kw. 553248

8 (Gerontolog* or Oldest Old or Elder* or Geriatric* or Senior* or Long-Lived or Over-the-hill or Senescen* or Centenarian or Nonagenarian or Octogenarian or Septuagenarian or Sexagenerian or Quinquagenarian or Unyoung).tw,kf,kw. 502358

9 ((Aged adj "50") or (Aged adj "60") or (Aged adj "70") or (Aged adj "75") or (Aged adj "80") or (Aged adj "85") or (Aged adj "90") or (Aged adj "95") or (Aged adj "100") or (Aged adj fifty*) or (Aged adj fifty-five) or (Aged adj sixty*) or (Aged adj sixty-five) or (Aged adj seventy*) or (Aged adj seventy-five) or (Aged adj eighty*) or (Aged adj eighty-five) or (Aged adj ninety*) or (Aged adj ninety-five) or (Aged adj one hundred) or (Aged adj a hundred)).tw,kf,kw. 67832

10 6 or 7 or 8 or 9 5992451

11 Women/ or Female/ 9675824

12 (Wom?n? or Female? or lady or ladies or maiden?).tw,kf,kw. 2498193

13 11 or 12 10123526

14 Cultural Diversity/ 12978

15 ((Diverse adj2 population?) or (Diverse adj2 culture?) or (Different adj2 culture?) or (intersection* adj3 identit*)).tw,kf,kw. 30214

16 (Diversity or Intersectionality).tw,kf,kw. 330298

17 14 or 15 or 16 366164

18 5 and 10 and 17 1203

19 5 and 13 and 17 2399

20 18 or 19 2639
